# Assessing the readability and quality of online written information on epistaxis

**DOI:** 10.1308/rcsann.2024.0053

**Published:** 2024-10-22

**Authors:** ZR Almansoor, R Abrar, H Raja

**Affiliations:** ^1^ The University of Manchester, UK; ^2^ Manchester University NHS Foundation Trust, UK; ^3^ University Hospitals Coventry and Warwickshire NHS Trust, UK

**Keywords:** Reading, Comprehension, COVID-19, Epistaxis, Nosebleeds

## Abstract

**Introduction:**

The objective of this study was to assess the readability and quality of online written information on epistaxis.

**Methods:**

The terms ‘epistaxis’ and ‘nosebleed’ were entered into Google. The first six webpages generated for each search term were screened. Readability was assessed using the Flesch–Kincaid Reading Ease Score (FRES), Flesch–Kincaid Grade Level (FKGL), Simple Measure of Gobbledygook (SMOG) Index and Gunning Fog Index (GFOG). Quality was assessed using the DISCERN instrument. Spearman’s correlation between quality and readability was calculated.

**Results:**

A total of 37 websites met the inclusion criteria. The mean and 95% confidence intervals for FRES, FKGL, SMOG and GFOG were 58.9 (55.3–62.5), 9.65 (8.74–10.6), 9.18 (8.57–9.8) and 12.5 (11.5–13.5), respectively. The DISCERN score was 34.3 (32.0–36.5). Weak negative correlation was noted between DISCERN and FRES (*r_s_* = −0.15, *p* = 0.36).

**Conclusions:**

Online information on epistaxis is generally of poor quality and low readability.

## Introduction

Epistaxis is one of the most common ear, nose and throat emergencies encountered by healthcare professionals.^1,2^ It carries a lifelong incidence of 60% across the general population, with the vast majority of cases being fairly self-limiting.^3^ A bimodal distribution is seen in the population, with incidence peaks at ages <10 years and above >50 years.^1^ Epistaxis most frequently occurs spontaneously, although it has a wide range of local, systemic, iatrogenic and environmental aetiologies, including digital manipulation and trauma.^2^ Initial management of epistaxis involves direct compression of the nasal alae for around 15min. Failure to control bleeding with compression warrants urgent medical review that may necessitate topical vasoconstrictors, silver nitrate cautery or anterior nasal packing.^4^ For more significant or recurrent epistaxis, posterior packing, arterial ligation or embolisation may be considered.^5^ Understanding the step-wise management approach to epistaxis is key to improving health outcomes and quality of life for patients, particularly among paediatric and elderly populations who can potentially experience anxiety and low mood.^6^

A growing number of patients are now using the internet to seek medical information.^7^ This trend has been accelerated through technological advancements and the COVID-19 pandemic, which has led to increased waiting times and reduced face-to-face appointments.^7,8^ Online healthcare information, however, often lacks accuracy and regulation, posing risks for patients' decision making and health-related outcomes.^9^

Although there has previously been an exploration of the quality of health information related to epistaxis on YouTube, there is currently a paucity of data on the appropriateness of online written health information in this field.^10,11^ This study aimed to address the gap by evaluating the readability and quality of online information on epistaxis.

## Methods

### Internet search methods

**Figure 1:**
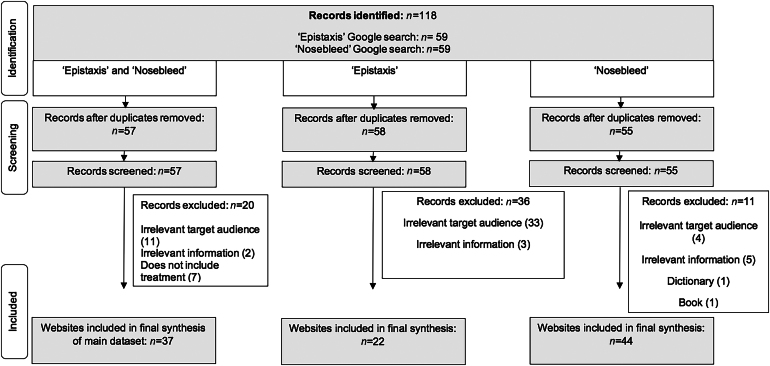
Flow diagram displaying the systematic search methodology. The searches were performed in July 2023.

The search terms ‘epistaxis’ and ‘nosebleed’ were entered separately into the Google search engine in July 2023. Cookies and browser history were deleted before the searches to eliminate interference from previous search results. The first six webpages for each search term were assessed for their readability and quality. This was to ensure a broad enough search scope because users are more likely to access only the first few Google webpages.^12^ Google was used as our main search engine owing to it occupying more than 90% of the overall market share worldwide.^13^ The workflow of our methodology is shown in Figure 1.

### Eligibility criteria

Our inclusion criteria consisted of websites that were accessible and written in English. Websites that provided irrelevant information or that did not discuss treatment for epistaxis were excluded. Other exclusion criteria included: if the target audience was healthcare professionals, if the word count was below the lower limit for readability scoring, and duplicate webpages.

All the websites were assessed objectively by two independent assessors (HR and ZA). Inconsistencies regarding the evaluation of a website were resolved by a third independent assessor who made the final decision.

### Readability assessment

The readability of each of the websites was assessed and scored using four validated scoring tools: Flesch–Kincaid Grade Level (FKGL), Flesch Reading Ease Score (FRES), Gunning Fog Index (GFOG) and the Simple Measure of Gobbledygook (SMOG) score.^14,15^ The scoring for each tool and how the reading difficulty is interpreted are shown in Table 1.

**Table 1:** Formulae and scoring of readability tools and their corresponding difficulty

Readability tool	Formula	Reading difficulty score interpretation
Flesch Reading Ease Score	206.835 − (1.015 × number of words/number of sentences) − (84.6 × number of syllables/number of words)	0–30: Very difficult 31–50: Difficult 51–60: Fairly difficult 61–70: Standard 71–80: Fairly easy 81–90: Easy 91–100: Very easy
Flesch–Kincaid Grade Level	0.39 × (average number of words per sentence) + (11.8×average number of syllables per word) − 15.59	College graduate: Very difficult College level: Difficult High school: Fairly difficult 8th-9th grade: Standard 7th grade: Fairly easy 6th grade: Easy 5th grade: Very easy
Gunning Fog Index	0.4 × [(words/sentences) + 100 (complex words/total words)]	17+: Very difficult 13–16: Difficult 10–12: Fairly difficult 8–9: Standard 7: Fairly easy 6: Easy 5: Very easy
Simple Measure of Gobbledygook	1.0430 √number of polysyllabic words + 3	17+: Very difficult 13–16: Difficult 9–12: Fairly difficult 8: Standard 7: Fairly easy 6: Easy 5: Very easy

Adapted from Boztas *et al*, Rees *et al* and De *et al*.^16–18^

### Quality assessment

The quality of online information was assessed using the DISCERN tool. This scoring tool uses 16 questions, divided into three sections, to evaluate the reliability and quality of information, through a five-point Likert scale. The first eight questions (Introduction) focus on the reliability of the information, the next seven questions (Methods) focus on details about treatment methods and the last question (Results) focuses on an overall rating of the information provided.^17^ Total DISCERN scores range from 16 to 80, with scores categorised as follows: very poor (16–29), poor (30–40), fair (41–51), good (52–63) and excellent (>64).

### Correlation assessment

Spearman's correlation was used to assess whether there was a correlation between quality (DISCERN scores) and readability (FRES scores).

### Statistical analysis

Statistical analyses were performed using Microsoft Excel® 2019. Statistical significance was set at *p* < 0.05. Mean and 95% confidence interval (CI) values for the readability and quality scores were generated for websites in the main data set. Statistical significance was set at *p* < 0.05.

## Results

A total of 118 websites were screened against the exclusion criteria. This resulted in 37 unique websites constituting the main data set (Figure 1).

### Readability assessment

Mean readability scores (95% CI) for FRES, FKGL, SMOG and GFOG were 58.9 (55.3–62.5), 9.65 (8.74–10.6), 9.18 (8.57–9.8) and 12.5 (11.5–13.5), respectively (Table 2). These scores are equivalent to reading ages of a 15–18 year old, 14–15 year old, 11–12 year old, and 17–18 year old, respectively.

**Table 2:** Summary of readability and quality data for ‘epistaxis’ and ‘nosebleed’ terms

Test name	Main data set search term analysis	Search term-specific subgroup analysis		
	‘Epistaxis’ and ‘nosebleed’ (*n* = 37)	‘Epistaxis’ (*n* = 22)	‘Nosebleed’ (*n* = 44)	*p*-value
Flesch Reading Ease Score mean (95% CI)	58.9 (55.3–62.5)	56.1 (51.8–60.4)	72 (51.3–92.7)	0.2955
Flesch–Kincaid Grade Level mean (95% CI)	9.65 (8.74–10.6)	10.1 (9.1–11.1)	9.4 (8.5–10.3)	0.3569
Simple Measure of Gobbledygook mean (95% CI)	9.18 (8.57–9.8)	9.5 (8.8–10.2)	8.8 (8.3–9.4)	0.1686
Gunning Fog Index mean (95% CI)	12.5 (11.5–13.5)	12.9 (8.4–10.6)	11.9 (10.9–12.9)	0.2379
DISCERN score mean (95% CI)	34.3 (32.0–36.5)	32.4 (29.6–35.2)	32.9 (30.8–35.0)	0.7803

*p* < 0.05 for significance. CI = confidence interval.

On sub-analysis of the search term ‘epistaxis’, the 22 websites had an average score (95% CI) of 56.1 (51.8–60.4), 10.1 (9.1–11.1), 9.5 (8.8–10.2), and 12.9 (8.4–10.6) for FRES, FKGL, SMOG and GFOG, respectively. These scores are equivalent to the reading ages of a 15–18 year old, 15–16-year-old, 11–12 year old and 17–18 year old, respectively.

On sub-analysis of the search term ‘nosebleed’, the 44 websites had an average score (95% CI) of 72 (51.3–92.7), 9.4 (8.5–10.3), 8.8 (8.3–9.4), and 11.9 (10.9–12.9) for FRES, FKGL, SMOG and GFOG, respectively. These scores are equivalent to the reading ages of a 12–13 year old, 14–15 year old, 11–12 year old and 16–17 year old, respectively.

### Quality assessment

The average DISCERN score and 95% CI when using the main data set of 37 websites were 34.3 (32.0–36.5). No websites had a score of good or excellent. Eight websites (22%) had a DISCERN score of fair and 10 (27%) had a score of very poor. Thirty-two websites (86%) had a poor/very poor reliability and 27 websites (73%) had a poor/very poor quality. One website (3%) had minimal deficiencies, whereas 31 websites (84%) had potentially important but not serious deficiencies.

When using the search term ‘epistaxis’, the average DISCERN score and 95% CI with regards to quality were 32.4 (29.6–35.2). Nineteen websites (86%) had a poor/very poor quality of information, with 16 (73%) having a poor/very poor reliability. Three websites (14%) had extensive deficiencies with the remainder (86%) having potentially important but not serious deficiencies.

When using the search term ‘nosebleed’, the average DISCERN score and 95% CI with regards to quality were 32.9 (30.8–35). Thirty-seven websites (84%) had a poor/very poor quality of information, with 34 (77%) having a poor/very poor reliability. Five websites (11%) had extensive deficiencies, with 38 (86%) having potentially important but not serious deficiencies.

### Correlation between FRES and DISCERN

**Figure 2:**
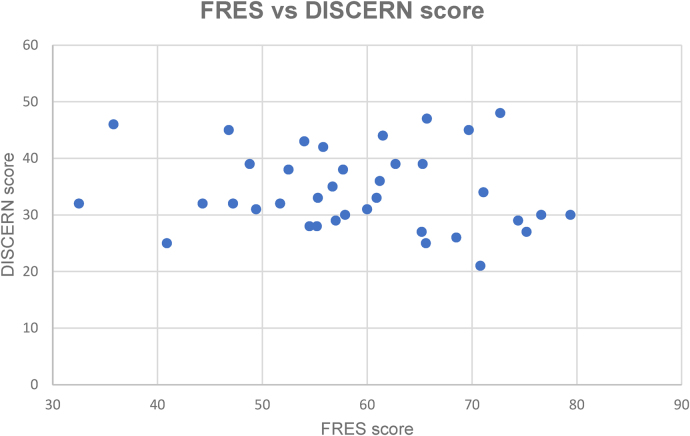
Scatter plot of Flesch Reading Ease Score (FRES) against DISCERN score for the main data set. Weak negative correlation was noted (*r_s_* = –0.15, *p* = 0.36).

There was a weak negative correlation between the FRES and DISCERN scores (*r_s_* = –0.15, *p* = 0.36; Figure 2).

## Discussion

Epistaxis is one of the most common ear, nose and throat emergencies. The majority of cases, however, do not require hospital treatment, with simple first aid measures providing therapeutic relief, often in the home setting.^2,3^ A few cases of epistaxis can be potentially life-threatening and compromise a patient's airway, breathing and circulation.^2,3^ Ensuring the public have access to accurate, relevant and easily comprehensible online material on epistaxis is therefore paramount. This is the first study, to our knowledge, to have assessed the readability and quality of online information on epistaxis. We found that information on epistaxis is generally of poor quality and written at a reading level beyond what the average person in the UK would be able to understand.

The readability of online information on epistaxis demonstrated that a large proportion of websites have a reading difficulty beyond the UK recommendation of 11 years old.^18^ All websites from our main data set had a reading age between 14 and 18 years, based upon FRES, FKGL and GFOG scoring. Given that the average person in the UK has a reading age between 9 and 15 years old, this suggests that online health material relating to epistaxis is generally too difficult to comprehend for the general population.^19,20^ This is concerning from a public health and economic perspective because poor readability levels can affect understanding of epistaxis management, resulting in poor adherence to first aid measures, delay in seeking medical attention and possibly increased hospital admissions.^21^

We found that no websites were at the recommended reading age based on FRES and GFOG scoring, with only two websites (5.4%) at the UK recommendation based on FKGL scoring. Our findings, however, based on SMOG scoring are contradictory to this, with 32 websites at a reading age of 11 years old. Because SMOG scoring is calculated based on the total number of polysyllabic words per sentence, caution is required as it may have slightly downplayed the overall readability difficulty. Unsurprisingly, we noted that using the search term ‘nosebleed’ instead of the search term ‘epistaxis’ generated websites that were slightly closer to the recommended UK reading age.

In terms of quality, the mean DISCERN score from our main data set equated to a poor level. This was mainly due to websites not making their aims clear, providing minimal information about the risks, benefits and options relating to the treatment of epistaxis, and offering limited sources of additional support. Omission of such details can negatively impact patient empowerment, resulting in patients having reduced responsibility over their healthcare decisions and actions. This can lead to possibly worse health outcomes for patients with epistaxis.

A weak negative correlation was found between the readability and quality scores for online information relating to epistaxis. Exploring this correlation is important because online materials need to be both easy to read and of high quality to improve patient health outcomes. Our data suggest that epistaxis information that is difficult to read is of higher quality, thus possibly impairing comprehension for some patients and, therefore, adversely affecting health outcomes. However, given that readability tools cannot assess the impact of website layout or the use of images, which can also influence the readability of online information, the exact correlation is difficult to determine. Despite this issue, our findings are in line with existing literature in otolaryngology, suggesting that concerted efforts are required to improve written health information for patients, irrespective of website layout and the use of images, and that using these tools are an appropriate method of assessing correlation.^22,23^

Although there is paucity of resources on epistaxis that are both easy to read and of high quality, we recommend that patients are directed to websites that currently best fit this remit, namely WebMD and The University Hospitals Sussex patient leaflet.^24^^,25^ Future online resources on epistaxis should ideally be produced by qualified healthcare professionals and clinicians who adopt NHS Digital guidelines while incorporating patient and public involvement. In addition, periodic expert review of online content should be undertaken to ensure timely updates are performed, including accurate reflection of the latest evidence base on epistaxis. This will ensure greater rigour, in terms of both readability and quality, and keep patients and the public better informed.

### Study limitations

Our study has some limitations. First, readability tools are unable to analyse audio-, image- or video-based information. These are key components that have the potential to make information more comprehensible and thus we may have underestimated the readability of some websites.^26^ Second, DISCERN scoring relies on a degree of subjectivity from the assessor, limiting the reproducibility of our findings. Although not undertaken in this study, interrater reliability could be used to assess level of agreement. Third, the internet is a dynamic platform, whereby search engines and their respective ranking algorithms may alter the order of search results.

## Conclusion

This study evaluated the quality and readability of online written information relating to epistaxis. Current online information on epistaxis is generally of poor quality and written beyond the UK recommended reading age. There is a weak negative correlation between the readability and quality of online written material for epistaxis. Healthcare professionals should be aware of these limitations and work actively to improve the readability and quality of online materials, while directing patients to high-quality, easily understandable resources.

## References

[1] National Institute for Health and Care Excellence (NICE). *Epistaxis*. https://cks.nice.org.uk/topics/epistaxis-nosebleeds/background-information/prevalence/ (cited July 2023).

[2] Kravchik L, Hohman MH, Pester JM. *StatPearls [Internet]*. Treasure Island, FL: StatPearls Publishing LLC; 2023. Anterior epistaxis nasal pack. https://www.ncbi.nlm.nih.gov/books/NBK538304/ (cited April 2024).

[3] Tunkel DE, Anne S, Payne SC *et al.* Clinical practice guideline: nosebleed (epistaxis). *Otolaryngol Head Neck Surg* 2020; **162**: S1–S38. 10.1177/0194599819890327 31910111

[4] Beck R, Sorge M, Schneider A, Dietz A. Current approaches to epistaxis treatment in primary and secondary care. *Dtsch Arztebl Int* 2018; **115**: 12–22. 29345234 10.3238/arztebl.2018.0012 PMC5778404

[5] Corr MJ, Tikka T, Douglas CM, Marshall J. One-year all-cause mortality for 338 patients admitted with epistaxis in a large tertiary ENT centre. *J Laryngol Otol* 2019; **133**: 487–493. 31062677 10.1017/S0022215119000860

[6] Huda MEB, Khalid AM, Hanaa SS. The impact of epistaxis on quality of life for primary school children in Dakahlia governorate, Egypt. *J Cardiovasc Dis Res* 2021; **12**.

[7] Hoehe MR, Thibaut F. Going digital: how technology use may influence human brains and behavior. *Dialogues Clin Neurosci* 2020; **22**: 93–97. 32699509 10.31887/DCNS.2020.22.2/mhoehe PMC7366947

[8] Vollmer MAC, Radhakrishnan S, Kont MD *et al.* The impact of the COVID-19 pandemic on patterns of attendance at emergency departments in two large London hospitals: an observational study. *BMC Health Serv Res* 2021; **21**: 1008. 34556119 10.1186/s12913-021-07008-9 PMC8460185

[9] Fahy E, Hardikar R, Fox A, Mackay S. Quality of patient health information on the Internet: reviewing a complex and evolving landscape. *Australas Med J* 2014; **7**: 24–28. 24567763 10.4066/AMJ.2014.1900 PMC3920473

[10] Devakumar H, Tailor BV, Perkins V, Ioannidis D. “How to stop a nosebleed”: a combined objective and subjective assessment of YouTube videos on first-aid management of epistaxis. *J Laryngol Otol* 2024; **138**: 169–177. 37409457 10.1017/S0022215123001184

[11] Haymes AT, Harries V. “How to stop a nosebleed”: an assessment of the quality of epistaxis treatment advice on YouTube. *J Laryngol Otol* 2016; **130**: 749–754. 27345303 10.1017/S0022215116008410

[12] Eysenbach G, Köhler C. How do consumers search for and appraise health information on the world wide web? Qualitative study using focus groups, usability tests, and in-depth interviews. *BMJ* 2002; **9**: 573–577. 10.1136/bmj.324.7337.573 PMC78994 11884321

[13] Statista. *Worldwide desktop market share of leading search engines from January 2010 to January 2022*. https://www.statista.com/statistics/216573/worldwide-market-share-of-search-engines/ (cited June 2023).

[14] Readability Formulas. *Readability scoring system*. https://readabilityformulas.com/free-readability-formula-tests.php (cited July 2023).

[15] Friedman DB, Hoffman-Goetz L. A systematic review of readability and comprehension instruments used for print and web-based cancer information. *Health Educ Behav* 2006; **33**: 352–373. 16699125 10.1177/1090198105277329

[16] Boztas N, Omur D, Ozbılgın S *et al.* Readability of internet-sourced patient education material related to “labour analgesia”. *Medicine (Baltimore)* 2017; **96**: e8526. 29137057 10.1097/MD.0000000000008526 PMC5690750

[17] Rees CE, Ford JE, Sheard CE. Evaluating the reliability of DISCERN: a tool for assessing the quality of written patient information on treatment choices. *Patient Educ Couns* 2002; **47**: 273–275. 12088606 10.1016/s0738-3991(01)00225-7

[18] De R, Pandey N, Pal A. Impact of digital surge during COVID-19 pandemic: a viewpoint on research and practice. *Int J Inf Manage* 2020; **55**. 10.1016/j.ijinfomgt.2020.102171 PMC7280123 32836633

[19] Boulos MN. British internet-derived patient information on diabetes mellitus: is it readable? *Diabetes Technol Ther* 2005; **7**: 528–535. 15929685 10.1089/dia.2005.7.528

[20] Gillies K, Huang W, Skea Z *et al.* Patient information leaflets (PILs) for UK randomised controlled trials: a feasibility study exploring whether they contain information to support decision making about trial participation. *Trials* 2014; **15**: 62. 24548781 10.1186/1745-6215-15-62 PMC3936815

[21] Safeer RS, Keenan J. Health literacy: the gap between physicians and patients. *Am Fam Physician* 2005; **72**: 463–468. 16100861

[22] Raja H, Lodhi S. Assessing the readability and quality of online information on anosmia. *Ann R Coll Surg Engl* 2023; **106**: 178–184. 37051757 10.1308/rcsann.2022.0147 PMC10830341

[23] Raja H, Almansoor ZR. Assessing the readability and quality of online information on benign paroxysmal positional vertigo. *Ann R Coll Surg Engl* 2023; **106**: 45–50. 36748797 10.1308/rcsann.2022.0150 PMC10757881

[24] WebMD. *Nosebleeds*. https://www.webmd.com/first-aid/nosebleeds-causes-and-treatments (cited July 2023).

[25] NHS University Hospitals Sussex. *Nosebleed (Epistaxis (emergency department leaflet))*. https://www.uhsussex.nhs.uk/resources/nosebleed-epistaxis-emergency-department-leaflet/ (cited July 2023).

[26] Badarudeen S, Sabharwal S. Assessing readability of patient education materials: current role in orthopaedics. *Clin Orthop Relat Res* 2010; **468**: 2572–2580. 20496023 10.1007/s11999-010-1380-y PMC3049622

